# Supplementary material for: A network-centric approach to drugging TNF-induced NF-κB signaling
Source: Nat Commun. 2019 Feb 26;10:860. doi: 10.1038/s41467-019-08802-0 (PMC6391473; doi:10.1038/s41467-019-08802-0)
Supplement: Supplementary file 1 — Supplementary Information [file 41467_2019_8802_MOESM1_ESM.pdf]

Supplementary Information

A network-centric approach to drugging TNF-induced NF-κB signaling

Pabon et al.

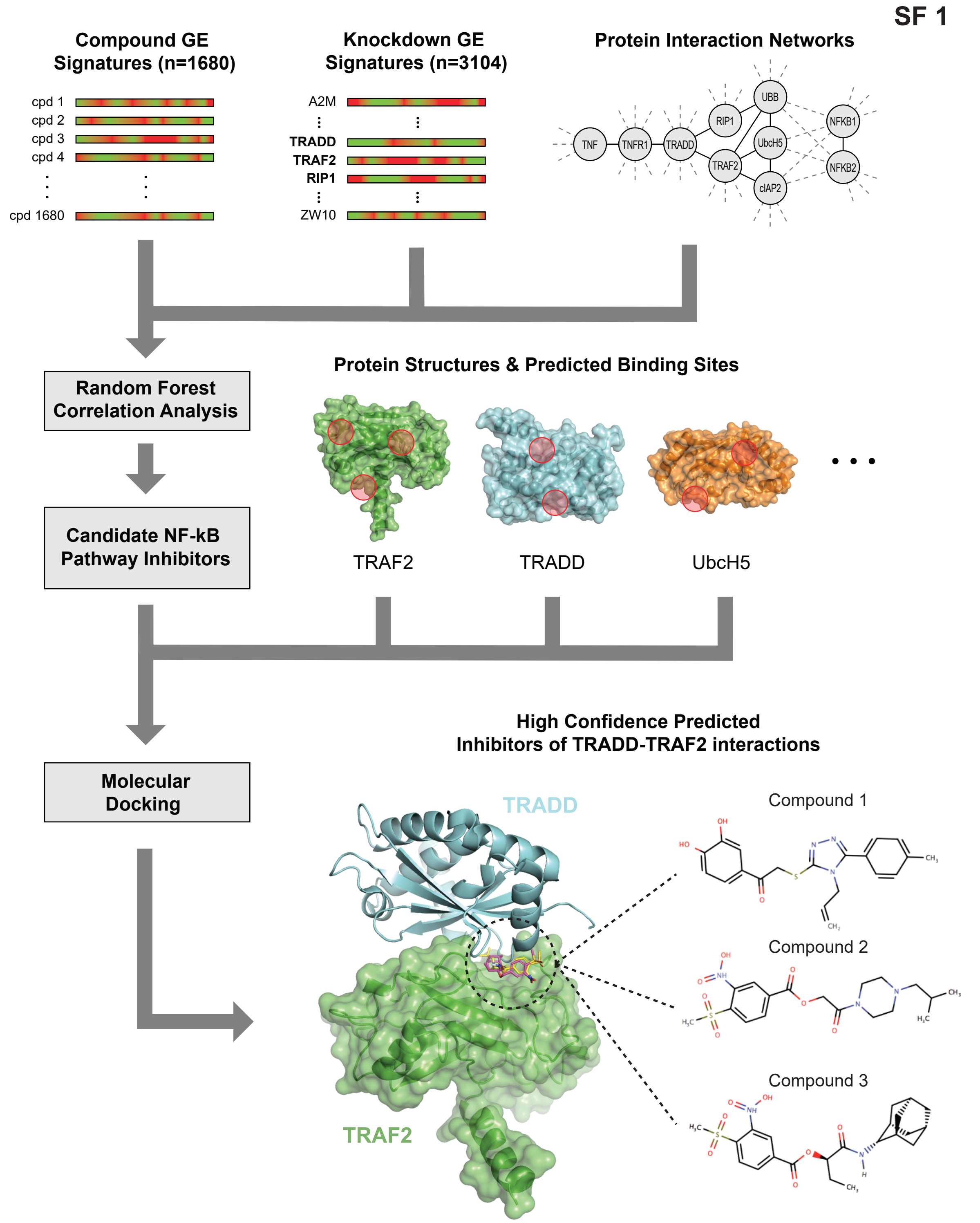

Supplementary Figure 1. Prediction pipeline used to identify small molecule inhibitors of TNF-inducible NF-kB signaling. Pipeline input includes cell-specific gene expression (GE) signatures from 1680 bioactive small molecules and 3104 gene knockdown, taken from the LINCS L1000 dataset[1], and the protein interaction networks of these genes, inferred from their BioGrid[2] interaction partners. Correlations between compound and knockdown GE signatures and their distribution on the TNF-inducible NF-kB pathway are evaluated by a random forest classifier to predict candidate inhibitors. Structural models of pathway proteins are mined from the PDB[3] and used as molecular docking targets for candidates. Docking results are assessed to identify high-confidence predicted inhibitors. Results are shown for TRADD-TRAF2 interactions in this study.

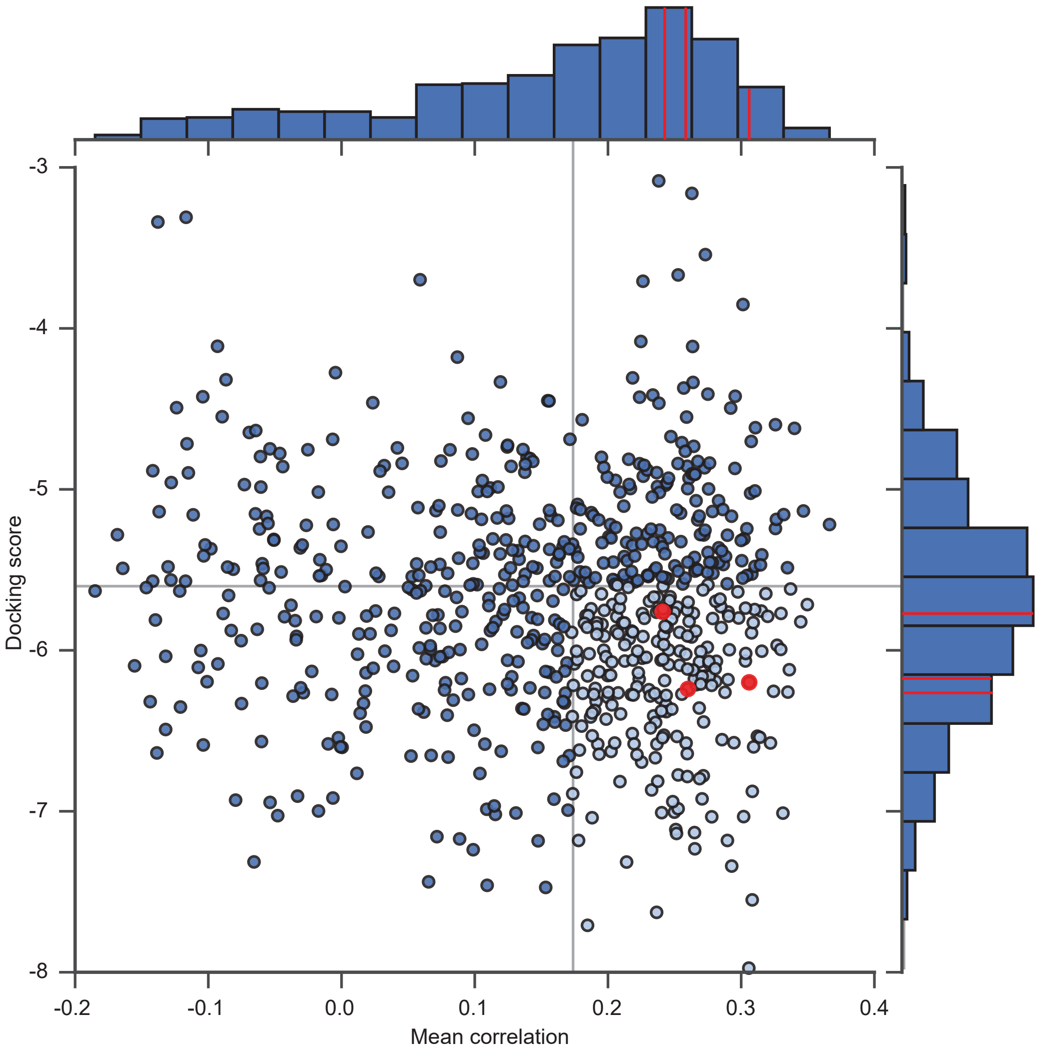

Supplementary Figure 2. Enrichment of NF-kB pathway disruptors. Horizontal axis shows the average Pearson correlation between the gene expression profile of the 717 compounds predicted to target TRAF2, TRADD or RIPK1[4] and the genes in the TNFR1 complex (Fig. 1a) present in the L1000 LINCS dataset (TRADD, TRAF2, TRAF5, UBB, UBC, RIPK1, TAB2, TAB3, UBE2D1, BIRC2, BIRC3, RBCK1, MAP3K7, IKBKB, CHUK, IKBKG). Vertical axis shows the Vina score (negative is better) of the docked poses[5] on the TRAF2-TRADD binding interface. Highlighted in red are the three compounds selected for testing that lie among 180 compounds in the top 50% of both axes. We note that many of the best Vina scores correspond to unusually large compounds with poor ligand efficiency[6]. Source data are provided as a Source Data file.

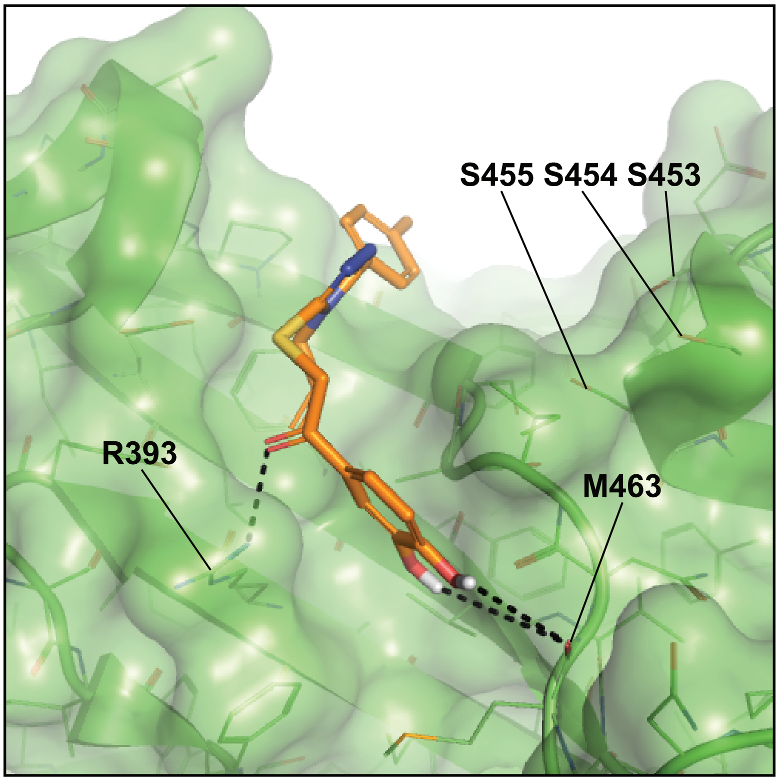

Supplementary Figure 3. Docking of compounds. Predicted binding mode of Compound 1 (orange) to TRADD-binding interface of TRAF2 (green). Hydrogen bonds are indicated with dotted lines.

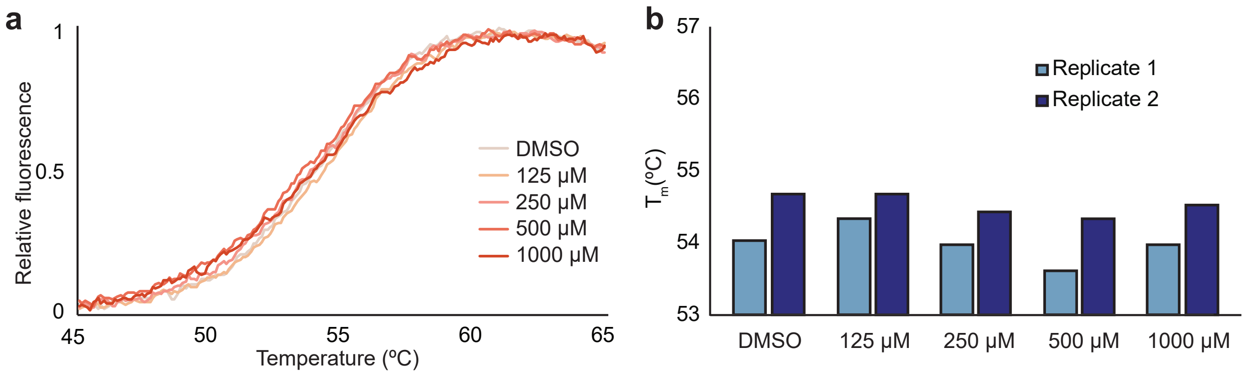

Supplementary Figure 4. Thermal shift assays indicate no clear effect of Compound 1 on TRAF2 stability. (a) Normalized melt curve of full length TRAF2 was recorded in the presence of DMSO or indicated concentrations of compound 1 (orange lines). **(b)** Melting temperature of TRAF2 in the present of compound 1 is not significantly altered in replicate experiments. Source data are provided as a Source Data file.

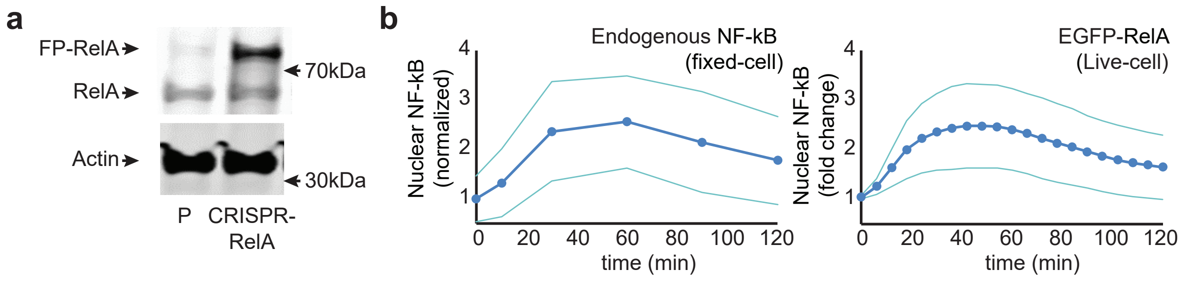

Supplementary Figure 5. Quantification of FP-RelA expression in U2OS cells. (a) Western blot of RelA in lysates from parental U2OS cells (P) and U2OS cells that were modified using CRISPR to express EGFP-RelA. The molecular weight of the dominant FP-RelA band in the CRISPR-modified cell line is shifted upward by 32 kDa, consistent with the expected molecular weight of the EGFP fusion protein. The presence of the wild type RelA band in the CRISPR-modified cell line suggests that only one allele of the RelA-encoding gene integrated the EGFP-encoding sequence. **(b)** Subcellular localization of RelA from fixed-cell immunofluorescence images of parental U2OS (left) and FP-RelA quantified from live cells using the CRISPR-modified cell line (right) exposed to 10 ng/mL TNF show similar temporal dynamics. Average of single cells (dark line) and standard deviation (light line) are shown. Source data are provided as a Source Data file.

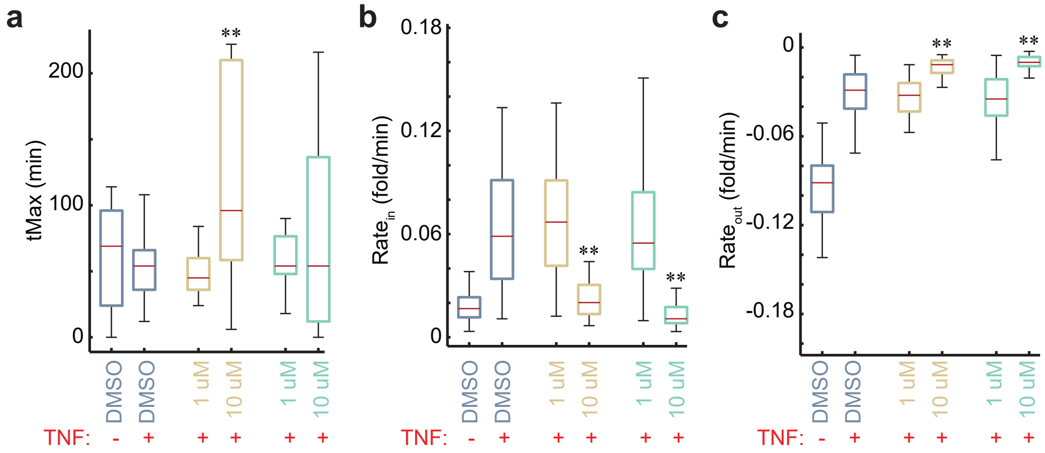

Supplementary Figure 6. Other descriptors of nuclear FP-RelA. Box (first and third quartile) and whisker (1.5 times interquartile range) plots for descriptors **(a)** t_{max}, **(b)** $Rate_{in}$, and **(c)** $Rate_{out}$ exposed to indicated conditions of TNF (10ng/mL) with either DMSO (blue), compound 2 (tan), or compound 3 (teal). Red centre line indicate the median. Double stars indicate statistically significant p-values (two-tailed) based on permutation test (see Supplementary figure 7). Red minus and plus symbols respectively indicate the absence or presence of TNF. Source data are provided as a Source Data file.

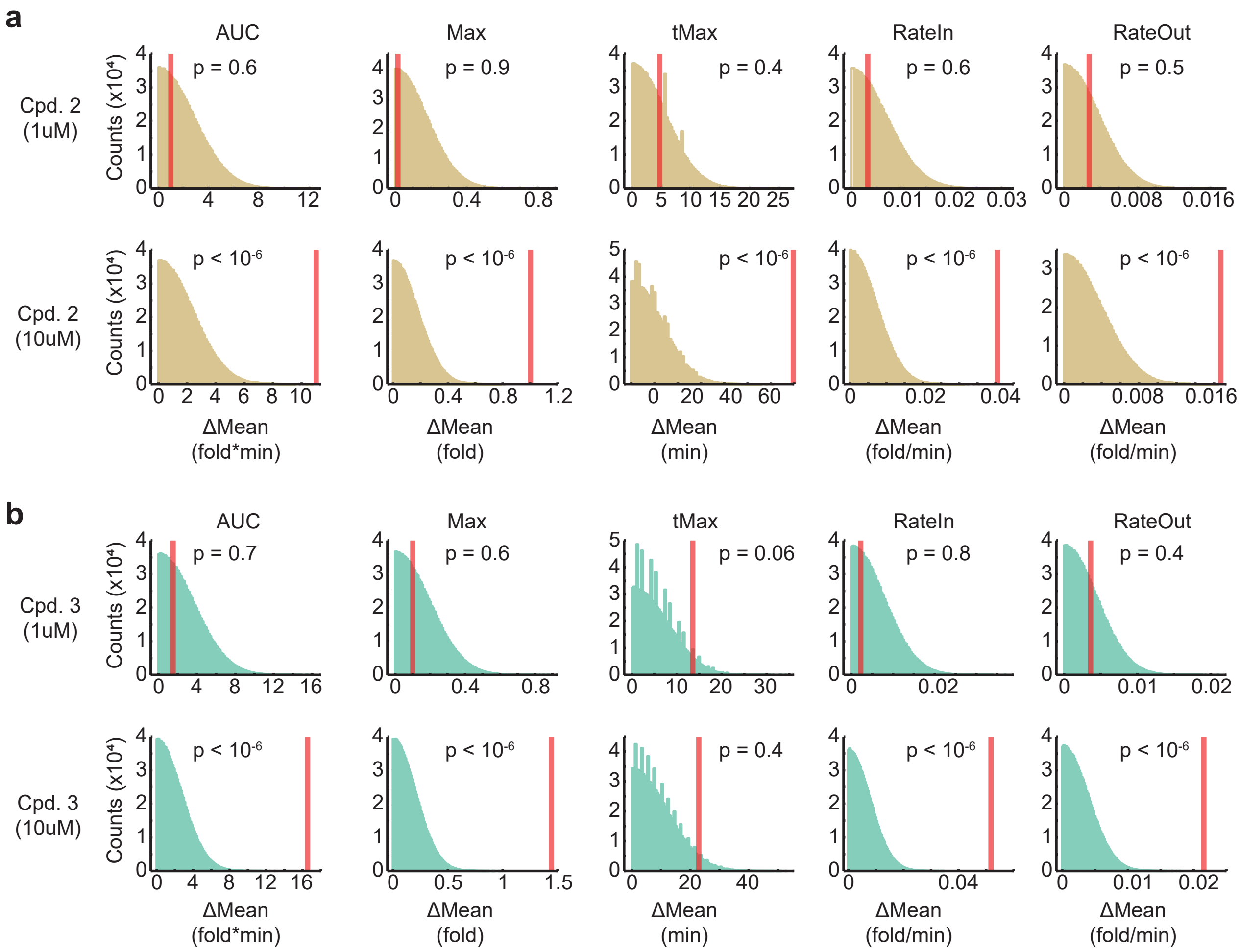

Supplementary Figure 7. Permutation tests for significance of nuclear FP-RelA descriptors. Histograms measuring the difference between the means for 10^6 permutations of data from the TNF-only control and TNF with indicated concentrations of **(a)** Compound 2 (tan distributions) and **(b)** Compound 3 (teal distributions). Red line indicates the difference between the means of un-permuted data and corresponding p-values (two-tailed) are listed for each permutation test.

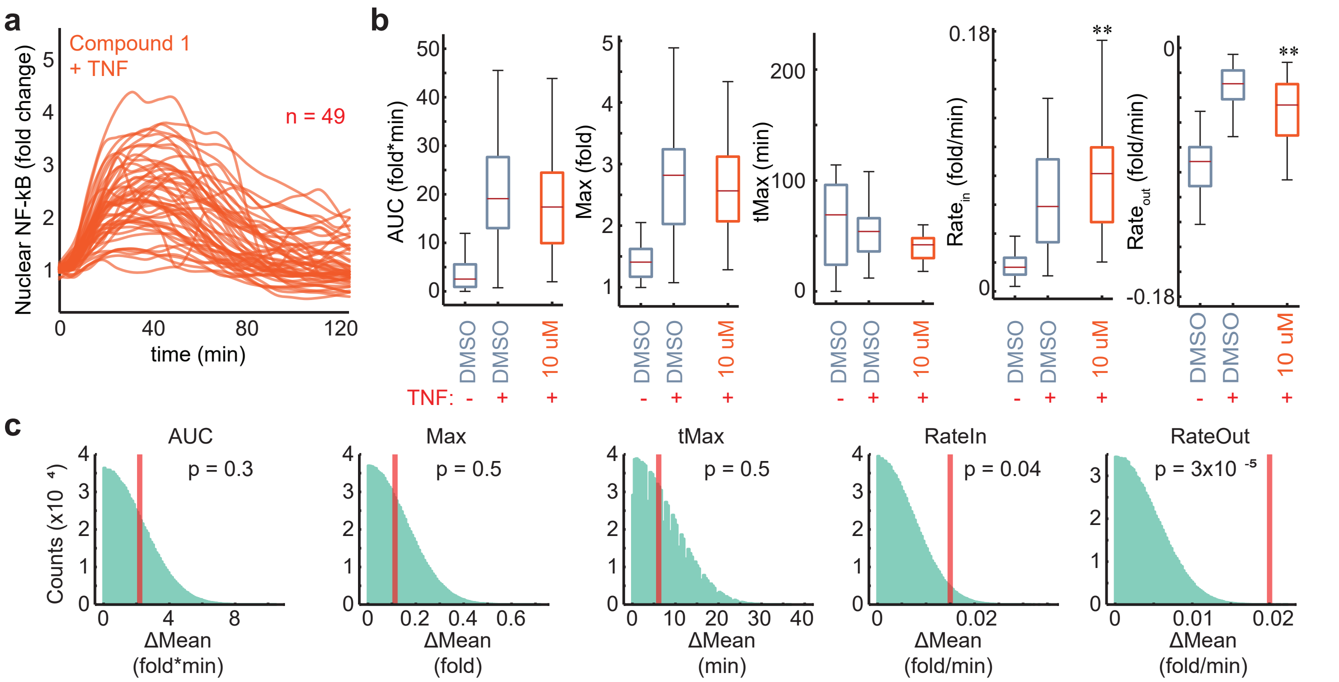

Supplementary Figure 8. Compound 1 does not have a significant effect on most descriptors of FP-RelA translocation. (a) Single cell time courses measure the change in nuclear abundance of FP-RelA in cells exposed to 10ng/mL TNF after pre-incubation with Compound 1. Red number indicates the number of single-cell trajectories. **(b)** Most descriptors of nuclear FP-RelA dynamics in panel (a) do not change significantly even in the presence of a high concentration of Compound 1. Box (first and third quartile), whisker (1.5 times interquartile range), and median (red centre line) are shown for cells pre-treated with DMSO (blue) or compound 1 (orange). Red minus and plus symbols respectively indicate the absence or presence of TNF. Double stars indicate statistically significant p-values based on permutation test shown in (c). **(c)** Histograms measuring the difference between the means for 10^6 permutations of data from the TNF-only control and 10uM of Compound 1. Red line indicates the difference between the means of un-permuted data and corresponding p-values (two-tailed) are listed for each permutation test. Source data are provided as a Source Data file.

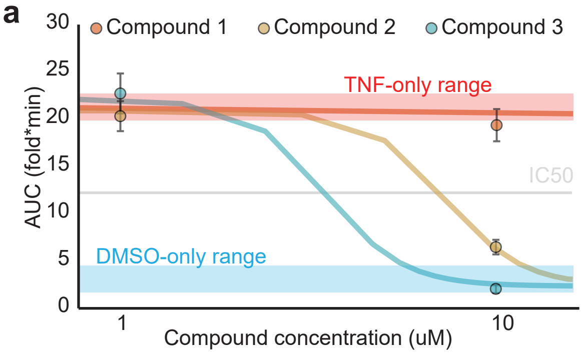

Supplemental figure 9. Dose-dependent inhibition of nuclear NF- kB dynamics. Plots from single cell descriptors data in Figure 3 for the **(a)** 'Area Under the fold change Curve' and **(b)** the 'Maximum Fold change'. For each descriptor, the response to TNF-only defines the upper limit of the average cellular response (top red band) and the response to DMSO only defines the lower limit (lower blue band). Plots of the mean and SEM of cellular responses in the presence of Compounds 1, 2, and 3 at the indicated concentration show that the IC50 for Compounds 1 and 2 is between 1 and 10 uM. Adjoining lines guide the eye across a hypothetical dose-response curve. Source data are provided as a Source Data file.

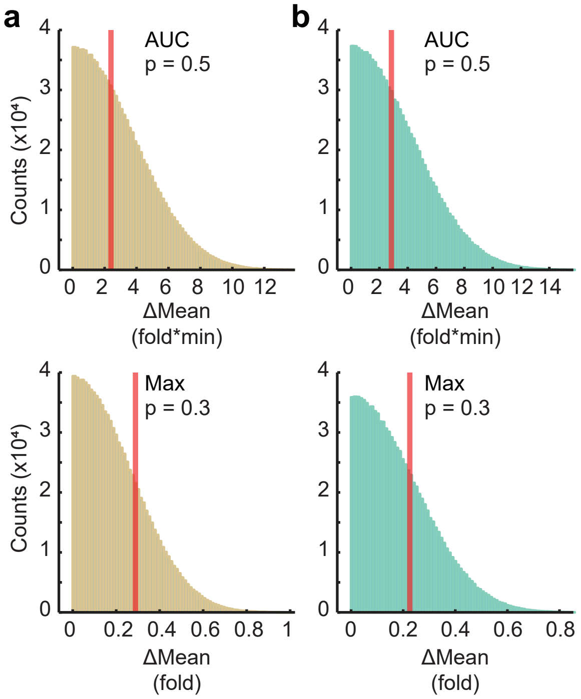

Supplementary Figure 10. Permutation tests for descriptors for dynamics of nuclear NF-kB in response to IL1. Histograms measuring the difference between the means of indicated descriptors for 10^6 permutations of data from the IL1-only control and IL1 in the presence of 10 uM of **(a)** Compound 2 (tan) and **(b)** Compound 3 (teal). Red line indicates the difference between the means of un-permuted data and corresponding p-values (two-tailed) for the permutation test demonstrate that the descriptors are not significantly altered.

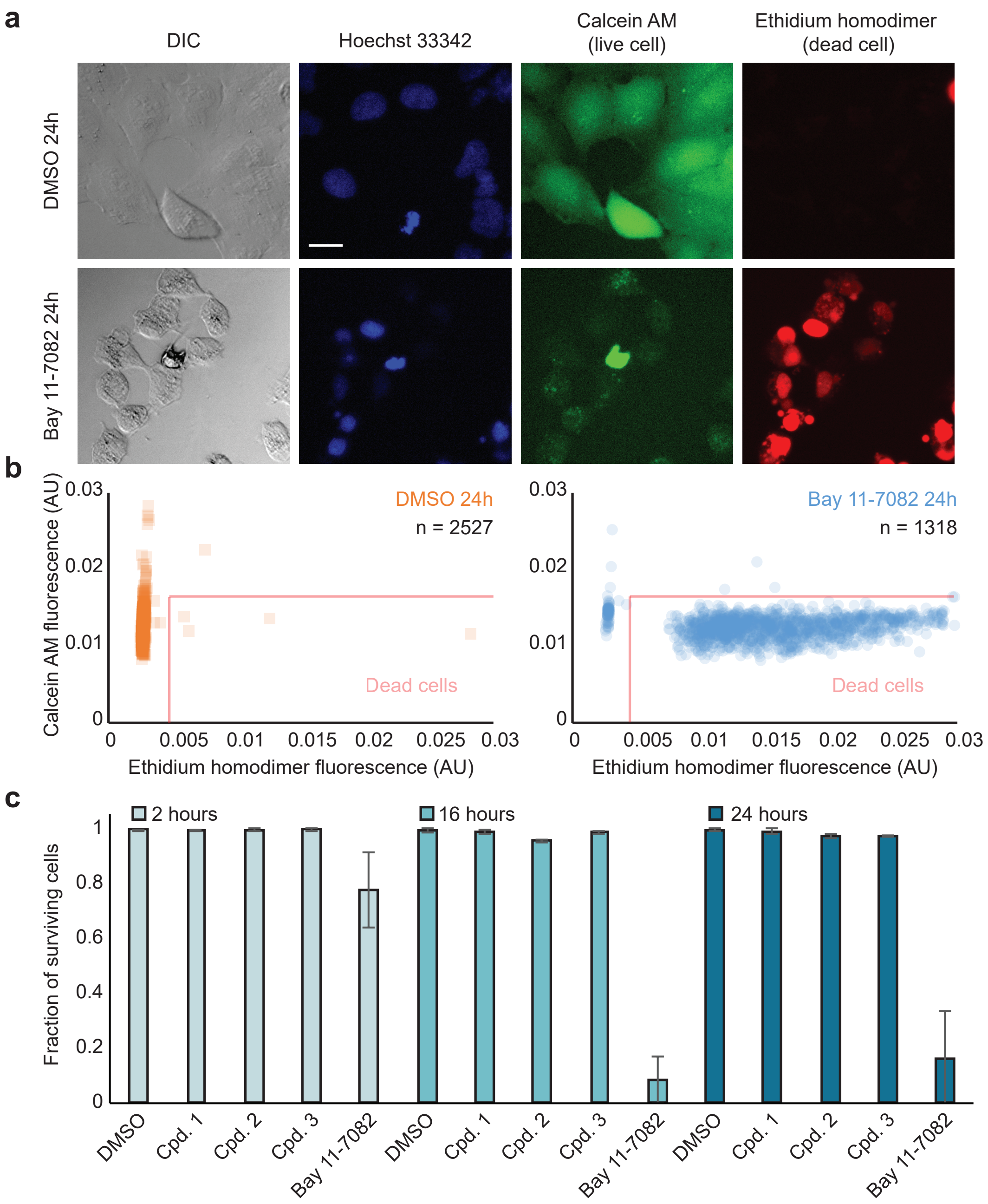

Supplementary Figure 11. Compounds have low cytotoxicity. (a) Representative live-cell images of cells stained with Calcein AM, a cell-permeable compound that becomes fluorescent only in viable cells, and Ethidium homodimer 1 which accumulates in the nucleus of dead cells only. Cells were exposed to either DMSO (top) or Bay 11-7082 (bottom) for 24 hours before imaging. Bay 11-7082 is a common NF-kB inhibitor with a mode of action that prevents activation of IKK kinases. Cells were also stained with Hoechst 33342 to assist with nuclear segmentation. Scale bar 20µm. **(b)** Example scatterplot for distributions of fluorescence measured in the nucleus of single cells exposed to DMSO (left, orange squares) or Bay 11-7082 (right, blue circles). Cells positive for Ethidium homodimer fluorescence and with low Calcein AM fluorescence were gated (red box) to identify dead cells. The remaining cells outside of the gate were considered alive. The fraction of surviving cells is quantified in **(c)** for the indicated duration of exposure conditions. Data shown for 3 biological replicates, ± SEM. On average, n=1600 single cells were measured per condition for each replicate. Source data are provided as a Source Data file.

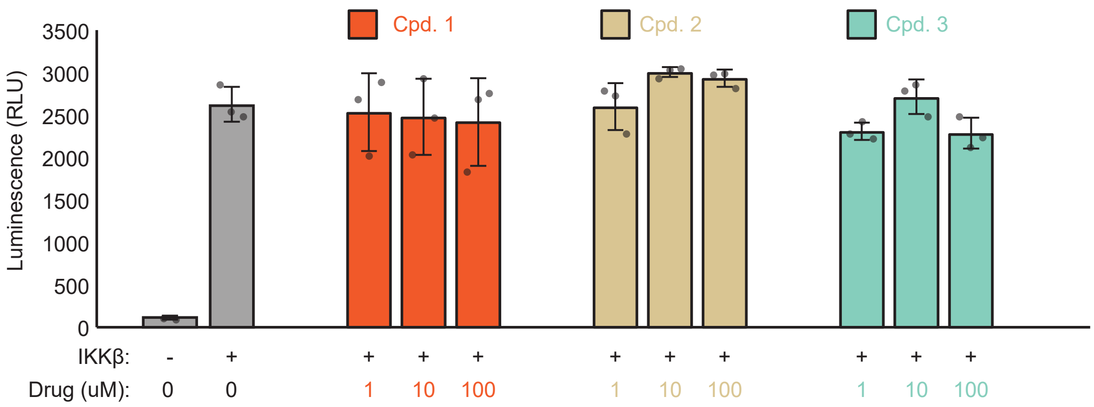

Supplementary Figure 12. Compounds do not inhibit IKKβ kinase activity. Luminescence-based *in vitro* kinase reactions using a recombinant activated IKKβ and a substrate peptide derived from human IκBα. For compounds 1 (orange), 2 (tan), and 3 (teal), kinase activity is not inhibited even at 10-fold higher concentrations than used in cell-based experiments. Minus and plus symbols respectively indicate the absence or presence of recombinant kinase in the reaction. Data shown for 3 replicate experiments (indicated by dot plots), ± standard deviation. Source data are provided as a Source Data file.

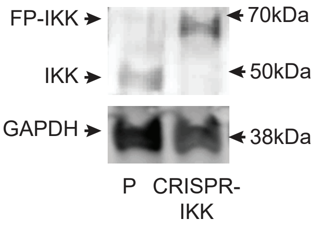

Supplementary Figure 13. Western blot of IKKγ. Western blot of IKKγ in lysates from parental U2OS cells (P) and U2OS cells that were modified using CRISPR to express EGFP-IKKγ. The molecular weight of the FP-IKKγ band in the CRISPR-modified cell line is shifted upward by 32 kDa, consistent with the expected molecular weight of the EGFP fusion protein. The absence of wild type IKKγ in the CRISPR-modified cell line suggests that both alleles of the IKKγ-encoding gene integrated the EGFP sequence. Source data are provided as a Source Data file.

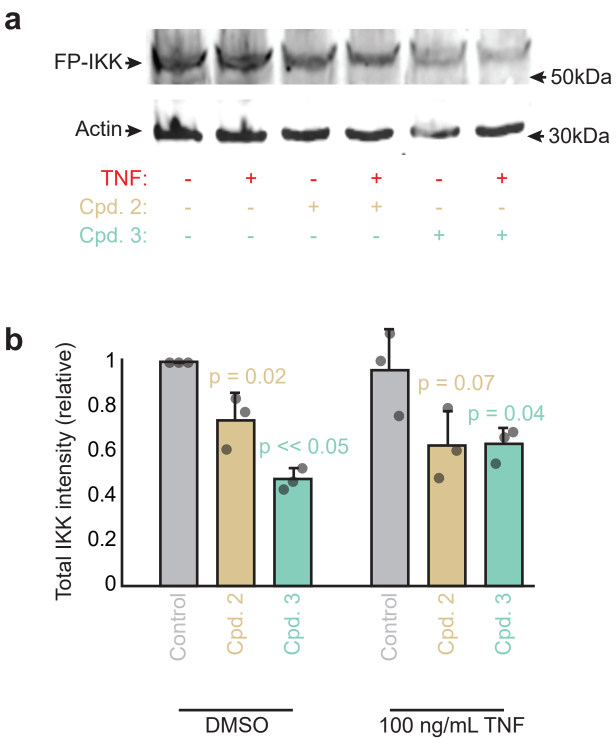

Supplementary Figure 14. IKKγ expression in the presence of compounds 2 and 3. (a) Western blot of IKKγ in lysates from CRISPR-modified U2OS cells in the indicated conditions. Minus and plus symbols respectively indicate the presence or absence for each of TNF (red), compound 2 (tan), and compound 3 (teal). **(b)** Quantification of Actin-corrected IKK band intensity, normalized to control cells that were not pre-treated with compounds (gray), suggest that the presence of compounds 2 (tan) and 3 (teal) downregulate the expression of IKKγ. Indicated p-values (two-tailed) calculated from t tests of biological triplicates (indicated by dot plots). ± standard deviation. Source data are provided as a Source Data file.

Compound 2

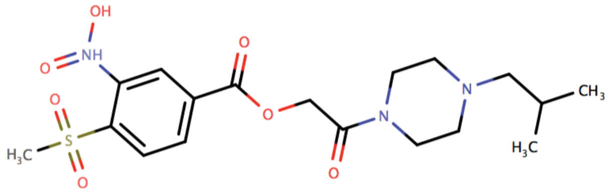

Compound 3

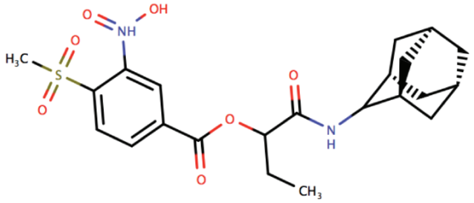

MolPort-004-495-831

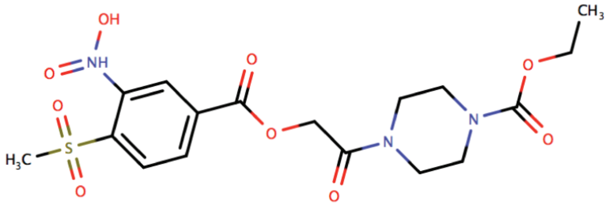

MolPort-004-588-414

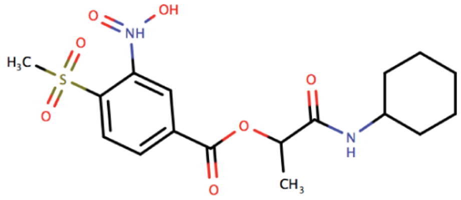

Supplementary Figure 15. Chemical structure of compounds and their commercially available versions. 1-isobutylpiperazine group in compound 2 was replaced by ethyl piperazine-1-carboxylate in Molprot-004-495-831. Bulkier adamantan-2-amine in compound 3 was replaced by cyclohexane in Molprot-004-588-414. Note that the modified groups do not participate in any of the key interactions predicted in Fig. 1c, and the changes are almost exclusively on surface/solvent areas.

References

1 Keenan, A. B. *et al.* The Library of Integrated Network-Based Cellular Signatures NIH Program: System-Level Cataloging of Human Cells Response to Perturbations. *Cell Syst* **6**, 13-24, doi:10.1016/j.cels.2017.11.001 (2018).

2 Chatr-Aryamontri, A. *et al.* The BioGRID interaction database: 2015 update. *Nucleic Acids Res* **43**, D470-478, doi:10.1093/nar/gku1204 (2015).

3 Berman, H. M. *et al.* The Protein Data Bank. *Nucleic Acids Res* **28**, 235-242 (2000).

4 Pabon, N. A. *et al.* Predicting protein targets for drug-like compounds using transcriptomics. *PLoS Comput Biol* **14**, doi:10.1371/journal.pcbi.1006651 (2018).

5 Ye, Z., Baumgartner, M. P., Wingert, B. M. & Camacho, C. J. Optimal strategies for virtual screening of induced-fit and flexible target in the 2015 D3R Grand Challenge. *J Comput Aided Mol Des* **30**, 695-706, doi:10.1007/s10822-016-9941-0 (2016).

6 Kuntz, I. D., Chen, K., Sharp, K. A. & Kollman, P. A. The maximal affinity of ligands. *Proc Natl Acad Sci U S A* **96**, 9997-10002 (1999).
