# Supplementary material for: A network-centric approach to drugging TNF-induced NF-κB signaling
Source: Nat Commun. 2019 Feb 26;10:860. doi: 10.1038/s41467-019-08802-0 (PMC6391473; doi:10.1038/s41467-019-08802-0)
Supplement: Supplementary file 2 — Description of Additional Supplementary Files [file 41467_2019_8802_MOESM2_ESM.docx]

**Description of Additional Supplementary Files**

File Name: Supplementary Movie 1

Description: Nuclear translocation of FP-RelA in response to TNF. U2OS cells modified by CRISPR to express FP-RelA from its endogenous gene locus in response to 10 ng/mL TNF.

File Name: Supplementary Movie 2

Description: Transient localization of FP-IKK to puncta in response to TNF. U2OS cells modified by CRISPR to express FP-IKKγ from its endogenous gene locus in response to 10 ng/mL TNF.
